# Cancer interception by interceptor molecules: mechanistic, preclinical and human translational studies with chlorophylls

**DOI:** 10.1186/s41021-021-00180-8

**Published:** 2021-03-06

**Authors:** Roderick H. Dashwood

**Affiliations:** 1 Center for Epigenetics & Disease Prevention, Texas A&M Health, 2121 West Holcombe Blvd, Houston, TX 77030 USA; 2 Department of Translational Medical Sciences, Texas A&M College of Medicine, Houston, TX USA

**Keywords:** Antimutagen, Apoptosis, Cancer interception, Desmutagens, Molecular complexes, Ribonucleotide reductase

## Abstract

Before ‘cancer interception’ was first advocated, ‘interceptor molecules’ had been conceived as a sub-category of preventive agents that interfered with the earliest initiation steps in carcinogenesis. Three decades ago, a seminal review cataloged over fifty synthetic agents and natural products that were known or putative interceptor molecules. Chlorophylls and their derivatives garnered much interest based on the potent antimutagenic activity in the Salmonella assay, and the subsequent mechanistic work that provided proof-of-concept for direct molecular complexes with planar aromatic carcinogens. As the ‘interceptor molecule’ hypothesis evolved, mechanistic experiments and preclinical studies supported the view that chlorophylls can interact with environmental heterocyclic amines, aflatoxins, and polycyclic aromatic hydrocarbons to limit their uptake and bioavailability in vivo. Support also came from human translational studies involving ultralow dose detection in healthy volunteers, as well as intervention in at-risk subjects. Antimutagenic and antigenotoxic effects of natural and synthetic chlorophylls against small alkylating agents also highlighted the fact that non-interceptor mechanisms existed. This gave impetus to investigations broadly related to free radical scavenging, anti-inflammatory effects, immune modulation and photodynamic therapy. Therapeutic aspects of chlorophylls also were investigated, with evidence for cell cycle arrest and apoptosis in human cancer cells. As the science has evolved, new mechanistic leads continue to support the use and development of chlorophylls and their porphyrin derivatives for cancer interception, beyond the initial interest as interceptor molecules.

## Background

Long before the term 'cancer interception' came into vogue [1, 2], 'interceptor molecules' already had been conceptualized [3], incorporating 'desmutagens' and inhibitors that might prevent the formation of carcinogens [4–8]. Harman and Shankel [3] noted that antimutagens could act at multiple levels, including the following: (i) prevention of mutagen formation; (ii) interception of mutagens via cellular or tissue organization; (iii) interception of mutagens by metabolites or enzymes present in cells; (iv) neutralization or removal of pre-mutagenic lesions in DNA by chemical compounds; and (v) activation of mechanisms that enhanced error-free DNA repair, blocked error-prone DNA repair, or augmented the metabolic inactivation of mutagens. The diverse range of compounds surveyed included *N*-acetyl-L-cysteine, acylglucosylsterols, albumins, allyl sulfides, *p*-aminobenzoic acid, aromatic isothiocyanates, ascorbic acid, bilirubin and biliverdin, bioflavonoids, butylated hydroxyanisole and butylated hydroxytoluene, caffeic acid, calcium, L-carnosine, β-carotene, catechins, chalcones, chlorogenic acids, creatine and creatinine, curcumin, diallyl sulfides, α- and β-dicarbonyls, dithiolthiones, ellagic acid, eugenol, fatty acids, ferulic acid, fiber, gallic acid, *γ*-glutamylcysteine, glutathione, L-histidine, hydroxychavicol, hypotaurine, imidazole-4-acetate, mucins, myricetin, ovothiols, pantetheine, polyamines, polyphenols, polyunsaturated fatty acids, quercetin, retinoids, tannins, taurine, thiols, tocopherols, uric acid, vitamins, and miscellaneous other agents [3]. The latter review also included chlorophylls as putative interceptor molecules [3], as these phytochemicals will be discussed here in greater detail, given that the field has continued to evolve over the intervening three decades.

## Review

### Chlorophylls as interceptor molecules

Hayatsu and colleagues first reported on the antimutagenic activities of chlorophylls and other porphyrins in the Salmonella mutagenicity assay [9–11], which subsequently was confirmed by others [12–15]. A seminal report [10] described molecular complex formation in vitro between a heterocyclic amine mutagen and chlorophyllin (CHL), the water-soluble derivative of natural chlorophyll *a* (Chl*a*). Although evidence was lacking in a preclinical cancer model, important groundwork had been laid for the ‘interceptor molecule’ hypothesis, with the possibility that molecular complexes might lower carcinogen uptake and systemic bioavailability after oral exposure. The appeal of such a mechanism immediately was apparent, given the ubiquitous presence of spinach and other chlorophyll-rich green leafy vegetables in the human diet [16–18]. Moreover, the mechanism implied broad applicability against a wide range of planar aromatic carcinogens that were known from the literature, including cooked meat heterocyclic amines, environmental polycyclic aromatic hydrocarbons, and dietary aflatoxins [9–15]. These aspects will be reviewed in the following sections.

### Interception of heterocyclic amines by chlorophylls

Spectrophotometric titration studies provided evidence for molecular complex formation between CHL and 2-amino-3-methylimidazo [4,5-*f*] quinoline (IQ), as well as with a dozen other dietary heterocyclic amine mutagens [19–22]. An isosbestic point indicated the presence of a 1:1 complex in some cases, which was corroborated via the mole ratio plot, whereas other interactions were more consistent with 2:1 stoichiometry. Dissociation/binding constants and docking scores in silico supported reversible complexes involving multiple π-π (stacking) interactions stabilized by van der Waals and electrostatic bonds, which inhibited IQ-DNA binding in vitro [19]. An inverse correlation was observed between the binding constant (*K*b) of the complex and the antimutagenic potency in the Salmonella assay, i.e.*,* the more stable the interaction, the lower the concentration of CHL needed to inhibit mutagenicity [20]. Notably, these findings were extended to natural chlorophylls, chlorins, tetrapyrroles, and other porphyrins [21].

Based on the hypothesis that molecular complexes would limit carcinogen uptake and bioavailability, corroborative experiments were conducted in vivo [23–25]. Co-administration of CHL and ^14^C-labeled IQ to the rat via single oral gavage inhibited IQ-DNA adduct formation significantly in the liver and colon [23, 24], and IQ-derived radiolabel was reduced in liver, bile and urine while being increased simultaneously in the feces. Co-injection of CHL with ^14^C-labeled IQ into isolated loops of intestine in situ also reduced the absorption of IQ from the gut, compared with IQ alone [23]. Pretreatment with CHL in the drinking water for 2 days before administering IQ by single oral gavage increased the excretion of conjugated (detoxified) mutagens in the urine and feces [25]. Finally, administration of IQ three times per week by oral gavage, while providing CHL in the drinking water, inhibited significantly the total tumor burden at 52 weeks in the male F344 rat [26].

**Fig. 1:**
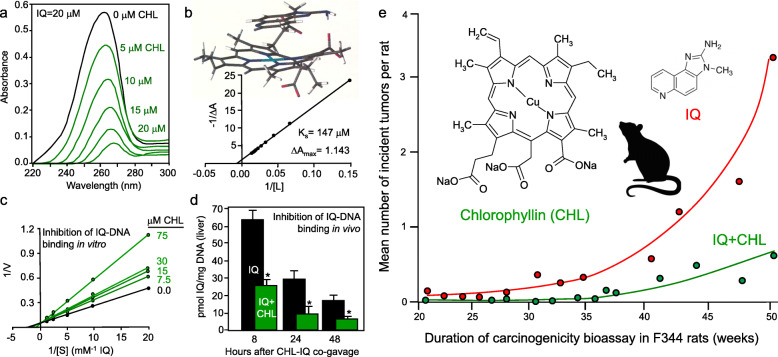
Chlorophyllin (CHL), a water-soluble derivative of natural chlorophyll, is an interceptor molecule of heterocyclic amines. **a** Spectrophotometric titration studies identified molecular complex formation between CHL and the cooked meat mutagen 2-amino-3-methylimidazo [4,5-*f*] quinoline (IQ). **b** Benesi-Hildebrand plot and molecular docking in silico of the CHL-IQ complex. Dissociation constants also were defined for other dietary heterocyclic amines, with complexes involving 1:1 or 2:1 stoichiometry. **c** In vitro, the kinetics of IQ-DNA binding indicated competitive inhibition by CHL. **d** In vivo, co-administration of CHL and IQ by single oral gavage reduced IQ-DNA adducts significantly in the rat liver (*P < 0.05). **e** Inhibition of IQ-induced tumorigenesis in the rat following co-treatment with CHL and IQ for 1 year. From the author’s published reports [19–26]

This was the first evidence in support of the anticarcinogenic activity of CHL in vivo, together with the work by Hasegawa et al. on the inhibition of mammary carcinogenesis in female rats treated with the heterocyclic amine 2-amino-1-methyl-6-phenylimidazo [4,5-*b*] pyridine (PhIP) [27]. Findings from the author’s laboratory are presented to highlight key aspects of the discussion above (Fig. 1).

### Interception of aflatoxins by chlorophylls

In addition to its potent antimutagenic activity towards aflatoxin B_1_ (AFB_1_), CHL also was highly effective against the direct-acting intermediate AFB_1_–8,9-epoxide in the absence of a metabolic activation system [13, 14]. Molecular docking in silico and spectrophotometric titration experiments supported a dissociation constant of *K*d = 1.4 ± 0.4 μM for the AFB_1_-CHL complex. In rainbow trout, AFB_1_-CHL coadministration in the diet produced concentration-dependent inhibition of AFB_1_-DNA adducts in the liver at 2 weeks by CHL, and predicted precisely the reduced incidence of hepatocellular carcinoma 9 months later [28–31].

Notably, hepatic AFB_1_-DNA adducts were reduced significantly in the rat and rainbow trout following simultaneous oral gavage administration of the carcinogen with either CHL or natural Chl*a* [31, 32]. The inhibition of multiorgan carcinogenesis occurred independently of changes in hepatic enzyme activities, supporting a mechanism involving complex-mediated reduction of carcinogen uptake in vivo [31, 32].

**Fig. 2:**
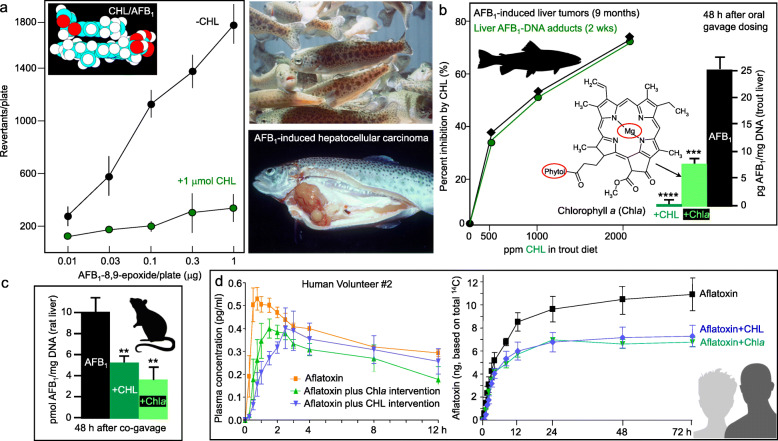
CHL and natural chlorophyll *a* (Chl*a*) act as interceptor molecules against aflatoxin B_1_ (AFB_1_). **a** Antimutagenic activity of CHL against AFB_1_–8,9-epoxide in the Salmonella assay, in the absence of a metabolic activation system, with complex formation supported by molecular docking in silico. **b** CHL inhibits AFB_1_-DNA binding and hepatocellular carcinoma in rainbow trout, and is more effective than Chl*a* at inhibiting AFB_1_-DNA adducts in trout liver (****P < 0.0001, ***P < 0.001). **c** CHL and Chla inhibit AFB_1_-DNA adducts in rat liver (**P < 0.01). **d** Accelerator mass spectrometry revealed that oral administration of CHL or Chl*a* both lowered AFB_1_ uptake and bioavailability in human volunteers. This work highlights the *continuum* from mechanistic to preclinical to human translational findings [14, 28–36]

In a landmark report [33], the bioavailability of aflatoxin was reduced significantly in human volunteers by CHL or natural Chl*a* isolated from spinach, using the sensitivity of accelerator mass spectrometry to detect microdosing levels in plasma and urine [34]. These findings extended prior work demonstrating that CHL intervention via an oral supplement reduced aflatoxin-DNA adducts in individuals at high risk for liver cancer [35, 36]. Highlights from the associated literature illustrate the *continuum* from mechanistic studies in vitro, to preclinical validation in animal models, and subsequent human translation (Fig. 2; silhouettes indicate trout, rat, and human translational aspects).

### Interception of polycyclic aromatic hydrocarbons by chlorophylls

Based on prior work [37] establishing the inhibitory actions of CHL and related pyrrole pigments against the mutagenicity of benzo [*a*] pyrene (BaP), detailed molecular mechanisms were investigated in vitro [15]. In the Salmonella assay, CHL inhibited the mutagenic activity of BaP in the presence of a metabolic activation system, and it was especially effective against the direct-acting ultimate carcinogen benzo [*a*]pyrene-7,8-dihydrodiol-9,10-epoxide (BPDE) in the absence of exogenous mammalian liver enzymes. Time-dependent hydrolysis of BPDE to inactive tetrols was observed in the presence of 5 μM CHL, whereas molecular complex formation with the procarcinogen (BaP) and the inhibition of cytochrome P450-related enzymatic activities necessitated higher CHL concentrations (> 100 μM). At these concentrations, CHL inhibited NADPH-cytochrome P450 reductase activity, rather than binding directly to the active site of cytochrome P450. Molecular models of the BPDE:CHL complex revealed minimization energies in the range − 16.9 to − 20.8 kcal/mol, with multiple π-π interactions of the overlapping aromatic ring systems, and the epoxide moiety of BPDE oriented towards acid (carboxyl) groups or the methylene bridge in CHL. It was concluded that the primary mechanism of CHL towards BaP in vivo involved preferential molecular complex formation with BPDE, leading to the rapid degradation of the ultimate carcinogen [15].

In female ICR mice, oral gavage dosing of CHL 30 min before either BaP or BPDE were given by topical administration resulted in significant inhibition of skin tumorigenesis, and CHL was rapidly distributed to the skin and other tissues [38]. The authors concluded that, under the experimental conditions used, the inhibition of skin carcinogenesis in mice was consistent with the interceptor molecule hypothesis.

**Fig. 3:**
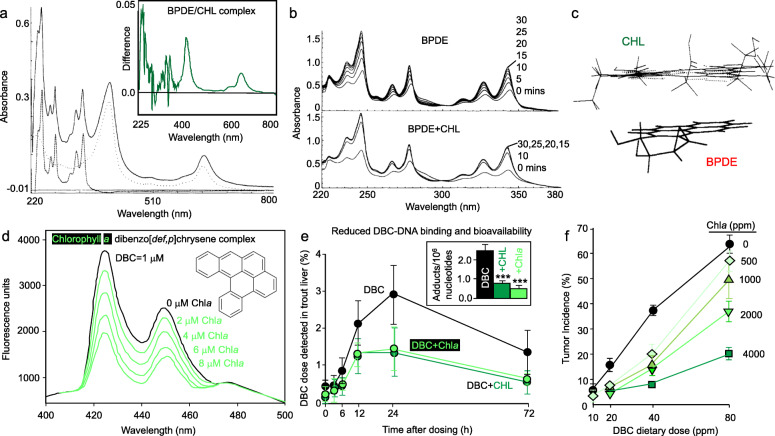
Chlorophylls as interceptor molecules of environmental polycyclic aromatic hydrocarbons. **a** Interaction of CHL with benzo [*a*]pyrene-7,8-dihydrodiol-9,10-epoxide (BPDE). Absorption spectra for BPDE (dashed line), CHL (dotted line), and BPDE+CHL (solid line) in Tris-HCl buffer, pH 7.4, 20 °C. Inset: difference spectrum for the BPDE/CHL complex. **b** Time-dependent hydrolysis of BPDE in presence and absence of CHL. Upper panel: the hydrolysis of 10 μM BPDE to tetrols was recorded at 5-min intervals in Tris-HCl buffer, pH 7.4, 20 °C. Lower panel: repeat of the above experiment in the presence of 5 μM CHL. Numbers adjacent to spectra indicate the time in minutes at which each spectrum was recorded. The pseudo-first-order hydrolysis rate constant of BPDE to tetrols was 1.76 ± 0.67 in buffer and 8.37 ± 0.82 in buffer containing CHL (*k*_H_ (s^− 1^) × 10^3^). **c** Energy minimized molecular model of the BPDE:CHL complex. Initial structures of CHL (top) and BPDE (bottom) were constructed using MM2 force field parameters and conjugate gradient methods. Complexes were obtained by first energy minimizing the structures of CHL and BPDE separately, then placing each molecule within Van der Waals radii and energy minimizing the corresponding complex. After multiple iterative docking experiments, complexes consistently had the epoxide oriented towards acid (carboxyl) groups or the methylene bridge in CHL, with minimization energies in the range − 16.9 to − 20.8 kcal/mol. Complexes were minimized to a gradient of 0.001 kcal/mol and calculations were performed using HyperChem (Release 2, Autodesk). **d** Natural Chl*a* complexes with dibenzo [*def,p*] chrysene (DBC). **e** Chl*a* and CHL lower the bioavailability of DBC and (inset) DBC-DNA adducts detected in trout liver (***P < 0.001). **f** Concentration-dependent inhibition of DBC-induced tumorigenesis in the rainbow trout. From published reports [15, 40–42]

Dibenzo [*def,p*] chrysene (DBC) – formerly known as dibenzo [*a,l*] pyrene – is a structurally-related environmental agent of BaP that has greater carcinogenic potency due to the presence of a ‘fjord’ region rather than a ‘bay’ region, which stabilizes the reactive epoxide intermediate [39]. In the rainbow trout, hepatic DBC-DNA adducts were inhibited significantly when the carcinogen was co-administered in the diet for 2 weeks with either CHL or natural Chl*a* [40]. Spectrophotometric titration studies supported a direct interaction between DBC and either CHL or Chl*a*. Thus, a 2:1 complex was observed for CHL-DBC, with *K*_d1_ = 1.38 μM and *K*_d2_ = 1.17 μM, whereas the 2:1 Chl*a*-DBC complex had *K*_d1_ = 4.44 μM and *K*_d2_ = 3.30 μM [41]. Pharmacokinetic data revealed that CHL and Chl*a* both lowered the systemic bioavailability of DBC to the liver and other tissues, consistent with the effective antitumor activity in this animal model [41, 42]. In the mouse, CHL provided effective chemoprotection in a DBC-induced transplacental carcinogenesis model, and supported a mechanism involving complex-mediated reduction of carcinogen uptake [43]. These findings suggest that additional studies are warranted on the beneficial effects of CHL and Chl*a* towards other environmental carcinogens and complex mixtures [44]. Mechanistic aspects for CHL and Chl*a* towards polycyclic aromatic hydrocarbons in vitro and in vivo were highlighted in Fig. 3.

### Non-interceptor mechanisms of chlorophylls

Despite the simplicity and attractive nature of the interceptor molecular hypothesis, it was known from early antimutagenicity work in vitro that CHL also was effective against *N*-methyl-*N*′-nitro-*N*-nitrosoguanidine [44]. This small alkylating agent lacks the aromatic ring system for stable molecular complexes involving multiple π-π interactions. Investigations with other small non-aromatic compounds, such as 1,2-dimethylhydrazine, azoxymethane and diethylnitrosamine, identified tumor modulatory activity even in post-initiation protocols, in which the carcinogen treatment phase had been completed before the addition of CHL or Chl*a* [45–55]. In some reports, the apparent tumor promoting activities may have been related to impurities in commercial CHL preparations [56, 57], which contained a mixture of sodium-potassium salts and chlorins [28], sometimes detectable in human plasma during clinical trials [35].

However, further investigation confirmed that CHL indeed exhibited mechanisms beyond molecular complex formation. The large tetrapyrrole macrocycle found in chlorophylls, chlorins, and other porphyrins has a high degree of unsaturation, which implicated free radical scavenging, antioxidant, anti-inflammatory, immune-modulatory and photodynamic properties. These aspects have been discussed previously for chlorophyllin and related porphyrins, including the effects on Wnt/β-catenin signaling and other deregulated pathways in cancer [58–76].

To examine mechanisms that go beyond molecular complex formation, human colon cancer cells were incubated with CHL across a broad range of concentrations [77, 78]. Fluorescence-activated cell sorting (FACS) analysis revealed that CHL-treated cells underwent S-phase arrest, and at higher concentrations a sub-G_1_ peak was detected, indicative of apoptosis [77]. Cells entering S-phase arrest exhibited a concentration-dependent loss of bromodeoxyuridine (BrdU) incorporation in FACS-based pulse-chase experiments. This was analogous to prior studies with chemotherapeutic drugs, such as hydroxyurea, that inhibited ribonucleotide reductase (RNR) activity by scavenging the tyrosyl radical involved in the catalytic mechanism [79–83]. Subsequently, CHL was confirmed to downregulate the expression of the large and small subunits of RNR, namely R1, R2 and p53R2, and to directly inhibit RNR enzymatic activity [78].

**Fig. 4:**
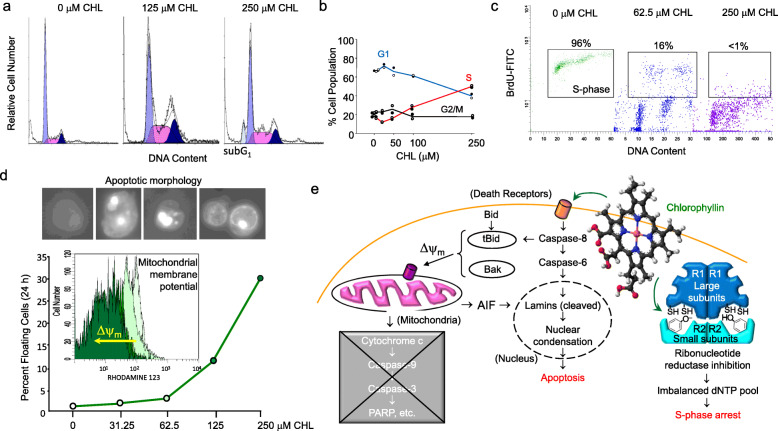
CHL triggers cell cycle arrest and apoptosis in human colon cancer cells. **a** In CHL-treated HCT116 cells, fluorescence-activated cell sorting (FACS) identified cells arrested in S-phase, with the appearance of a sub-G1 peak at higher CHL concentrations, indicative of apoptosis. **b** S-phase arrest by CHL was concentration dependent. **c** FACS analysis combined with bromodeoxyuridine (BrdU) pulse-chase experiments corroborated the S-phase arrest, with BrdU incorporation localized to G_0_/G_1_ and G_2_/M lower quadrants. **d** The floating cell number increased with CHL concentration, and cells exhibited hallmarks of apoptosis such as membrane blebbing and nuclear condensation, coinciding with reduced mitochondrial membrane potential (∆ψ_m_), arrow. **e** Detailed mechanistic studies excluded the pathway involving cytochrome *c*, Caspase-9, Caspase-3 and poly (ADP-ribose) polymerase (PARP) cleavage, and implicated Capase-8, Caspase-6, cleavage of nuclear lamins, and apoptosis inducing factor (AIF) released from mitochondria. At lower CHL concentrations, S-phase arrest involved the inhibition of ribonucleotide reductase, possibly by scavenging the tyrosyl radical in the enzyme active site, akin to the anticancer drug hydroxyurea. Synopsis of author’s prior mechanistic work [77, 78]

At higher concentrations, colon cancer cells exhibited the hallmarks of apoptosis, such as membrane blebbing and nuclear condensation, without the classical apoptotic ‘ladder’ in gel-based experiments [77]. Although a change in mitochondrial membrane potential was detected (∆ψ_m_), this was not accompanied by the release of cytochrome *c* or the activation of Caspase-9, Caspase-3, and poly (ADP-ribose) polymerase (PARP) cleavage. However, a cytochrome *c*-independent apoptotic mechanism was confirmed that involved Caspase-8, Caspase-6, the release from mitochondria of apoptosis inducing factor (AIF), and the cleavage of nuclear lamins [77]. This work advanced key concepts beyond prevention by an ‘interceptor molecule’ towards late-stage cancer interception, via mechanisms that impacted cell cycle regulation and apoptosis (Fig. 4).

Finally, epigenetic avenues remain to be pursued in vivo, beyond the downregulation of histone deacetylases in hamster-cheek pouch carcinomas by CHL [84], and the altered microRNA signatures in PhIP-induced rat colon tumors after feeding chlorophyll-rich spinach [85]. Antiviral aspects of CHL also are highly noteworthy [86].

## Conclusions

In conclusion, chlorophylls can act as interceptor molecules of environmental carcinogens and mutagens, but also exhibit additional mechanisms that impact the *continuum* of cancer initiation, promotion and progression. As the science evolves, interesting new mechanistic leads point to the potential use of chlorophylls and their derivatives for cancer interception, in its broadest context [1]. Thus, further research appears to be warranted, beyond the historical clinical applications and the current use of such natural and synthetic agents as food additives [87–90], directed towards environmental carcinogens and the human translational aspects [91–100].

## Data Availability

Not applicable – data are available in the public domain.

## Abbreviations

AFB_1_: Aflatoxin B_1_
AIF: Apoptosis inducing factor
BaP: Benzo [*a*]pyrene
BPDE: Benzo [*a*]pyrene-7,8-dihydrodiol-9,10-epoxide
BrdU: Bromodeoxyuridine
CHL: Chlorophyllin
Chl*a*: Chlorophyll *a*
DBC: Dibenzo [*def,p*]chrysene
FACS: Fluorescence-activated cell sorting
IQ: 2-amino-3-methylimidazo [4,5-*f*]quinoline
*K*b: Binding constant
*K*d: Dissociation constant
PARP: Poly (ADP-ribose)polymerase
PhIP: 2-amino-1-methyl-6-phenylimidazo [4,5-*b*]pyridine
RNR: Ribonucleotide reductase
